# Everybody's Page

**Published:** 1893-03-04

**Authors:** 


### EVERYBODY'S PAGE.

Large meetings of apothecaries are going to be held in Prague and Vienna for the purpose of addressing petitions to the Government in opposition to the scheme now afloat of admitting women to the career of chemists, which business, with a selfishness that may be pardoned, the petitioners prefer confined to their own sex.

The French in a hundred instances have turned the lesser things of this earth to good account. The thrift of a nation which can convert the vermin of its cities into objects of produce is worthy of our admiration, if not of our imitation. The town rat, which of all animals is generally considered the one most outside of our affections, is converted into a useful member of society in the Parisian capital. Here these creatures are collected together and placed in the great pound where the carcase refuse of the city is thrown. These remains are quickly demolished by the rats, who leave only untainted skeletons or bones behind them. The demolishers are in their turn destroyed themselves. Four times a year a great battue is effected, and when next the little creatures appear it is in the form of that article of world-wide admiration—the Gant de Paris; indeed, no skin is superior to theirs, the pliability and strength of it rendering it the most suitable for the glove market.

Bohemia is again furnishing us with an instance of miraculous appearance. It appears that in a small village near the Prussian frontier there lives a widow with her young daughter Christina. Christina had gone one day to gather mushrooms in a wood, when a strange lady dressed in black appeared to her there, spoke a few words, and departed. Later on the child met the same lady again—this time her clothes were grey. She spoke in a friendly manner, asking Christina to meet her again in five days' time. The young girl kept the appointment, and the strange visitant imparted to her the message with which she said she was burdened. "God's patience," she explained, "was exhausted; and I, my child," the lady said, "I have come to save His people." Christina besought some proof. "Would she cure the sick around?" she asked her heavenly friend; and in answer to this all those interceded for were cured of their illnesses. When this miraculous apparition was last seen it was in the sight of a multitude of 500 persons collected together to witness it. So runs the tale, which is thrilling the simple Bohemian folk with its wonders—and the explanation, if indeed any is forthcoming, has not been given yet.

There is a fashion in languages as much as there is a fashion in the mode of our clothes. Just now, English has the ascendancy, and is the dominant speech of the human race. Professor Vambéry, addressing the British Club the other evening at Budapest, computed the number of English-speaking men and women at 130 millions—a figure which, should it continue in the same proportion, will be nearly doubled by the middle of the coming century.

It is hardly admissible to classify all the inventions of the day under the heading of "Improvements." Under this latter category the new Electric Bath Chair may not yet be admitted; so far it is an invention only, and has not matured itself into an improvement on the old order of things. Electricity, which of all known forces is the one least subjective to human government, is barely the motive power best adapted for an invalid's use. Imagine a bath chair dashing in uncheckable impetus down a crowded esplanade, dragging its unfortunate inmate into certain perils of wheels and curb-stones; or imagine it uncertain in its method of progression, halting in some street crossing, and making a deadlock of all traffic. This chair, it seems to us, if indeed it is to be a success at all, will have to carry a certificated character along with it, signed at least by the patentee, the manufacturer, and two or more members of the County Council, to guarantee its being a chair of good faith, and in no way likely to carry out our fears on its behalf.

Much has been said, and most rightly too, of the injustice of our English Divorce Laws, wherein we find one standard of morality for the man, and another for the woman, but with all its faults marriage has not become in England purely a matter of geography, in which condition we may fairly say we find it in America. In that country it is quite possible for a man and a woman to be legally married in one State, and yet single in another through the difference in the laws of the different States. This system may be occasionally convenient in practice, but it is not very judicious in principle. The New Divorce Reform League, which has just held its annual meeting at Boston, has evidently recognized the necessity for a reform statute relating to the improvement of the American Marriage Laws. A State Commission is arranged for, to inquire further into the subject, and should the Reform League succeed in its admirable work, and satisfy the various territorial appeals, we shall no longer hear of the legal and illegal complications which have arisen from the present system of localised marriage in America.

